# Investigating pH based evaluation of fetal heart rate (FHR) recordings

**DOI:** 10.1007/s12553-017-0201-7

**Published:** 2017-07-04

**Authors:** George Georgoulas, Petros Karvelis, Jiří Spilka, Václav Chudáček, Chrysostomos D. Stylios, Lenka Lhotská

**Affiliations:** 1 Control Engineering Group Department of Computer Science, Electrical and Space Engineering Luleå University of Technology, SE-97187 Luleå, Sweden; 2 Laboratory of Knowledge and Intelligent Computing, Department of Computer Engineering, Technological Educational Institute of Epirus, Arta, Kostakioi Greece; 3 CIIRC, Czech Technical, University in Prague, Czech Republic, Prague, Czech Republic

**Keywords:** Fetal heart rate (FHR), Cardiotocography (CTG), Least Squares Support Vector Machines (LS-SVMs), Feature selection, Classification

## Abstract

Cardiotocography (CTG) is a standard tool for the assessment of fetal well-being during pregnancy and delivery. However, its interpretation is associated with high inter- and intra-observer variability. Since its introduction there have been numerous attempts to develop computerized systems assisting the evaluation of the CTG recording. Nevertheless these systems are still hardly used in a delivery ward. Two main approaches to computerized evaluation are encountered in the literature; the first one emulates existing guidelines, while the second one is more of a data-driven approach using signal processing and computational methods. The latter employs preprocessing, feature extraction/selection and a classifier that discriminates between two or more classes/conditions. These classes are often formed using the umbilical cord artery pH value measured after delivery. In this work an approach to Fetal Heart Rate (FHR) classification using pH is presented that could serve as a benchmark for reporting results on the unique open-access CTU-UHB CTG database, the largest and the only freely available database of this kind. The overall results using a very small number of features and a Least Squares Support Vector Machine (LS-SVM) classifier, are in accordance to the ones encountered in the literature and outperform the results of a baseline classification scheme proving the utility of using advanced data processing methods. Therefore the achieved results can be used as a benchmark for future research involving more informative features and/or better classification algorithms.

## Introduction

The aim of Cardiotocography (CTG) is to screen for signs of fetal distress thus allowing obstetricians to react in a timely fashion in order to prevent potential adverse outcomes for the fetus. CTG recordings, also referred to as Cardiotocograms, consist of the recording of two signals; the fetal heart rate (FHR) signal, measured in beats per minute (bpm) and the uterine contractions (UCs) signal, measured either in mmHg or in arbitrary units, and remain the only technique used world-wide that can provide continuous information about the state of the fetus during delivery [1, 2]. One of the reasons for the adoption of CTG, is the sense of “security” that it offers to the clinicians by providing on-line real time monitoring of the fetus. On the other hand, even though continuous information of the Cardiotocogram is an improvement over the previously used intermittent auscultation (IA), its evaluation is hindered by the large variance of the responses of individual fetuses to stress situations.

In the clinical practice the evaluation of Cardiotocograms primarily relies on eye inspection and assessment following guidelines, which usually stem from those issued by the International Federation of Gynecology and Obstetrics (FIGO guidelines [3]). Nevertheless, despite the existence of specific guidelines, Cardiotocogram interpretation suffers from high inter- and intra-observer variability among clinicians [4, 5]. Moreover, CTG is cited as one of the reasons behind the increase of the number of Caesarean sections [6]. On the other hand, since there are currently no significantly new approaches to fetal monitoring during delivery in the horizon, CTG is here to stay regardless of its flaws. To mitigate the variability in the evaluation of Cardiotocograms, two major ways have been proposed [7]: i) extensive training of the clinical staff or ii) use of computerized systems for decision support. In the latter case, the evaluation problem is usually cast as a classification one, where the classes for CTG evaluation are primarily based on umbilical artery cord blood analysis. The most common approach is to use the pH value with an *apriori* defined threshold to distinguish between acidotic and non-acidotic fetuses.

It should be noted, that the idea of developing computerized systems for Cardiotocogram evaluation is very old, even preceding the release of general FIGO guidelines [8]. Since these early works, many more approaches have been proposed, ranging from systems that emulate FIGO guidelines [9] to systems that rely on the extraction of features using advanced signal processing techniques coupled with advanced classification algorithms. Many of these features are based on the well-established adult Heart Rate Variability (HRV) research [10]; others come from the statistical analysis of the FHR [11], or from the nonlinear domain [12, 13]. Time-scale descriptors [14], features based on Empirical Mode Decomposition (EMD) [15] and even artificially generated features have also been proposed [16] and tested. Additionally, modeling of FHR and its behavior with relation to contractions has also been investigated [17]. For the classification part, methods such as Support Vector Machines (SVMs) [13, 14, 17–19], Artificial Neural Networks (ANNs) [11, 20–22], Generative Models (GMs) [23], Fuzzy Systems [24, 25], and Hidden Markov Models (HMMs) [26] have been tested among other computational systems.

Regarding the studies that use pH values to define the respective classes, in [23] different GMs are trained using features calculated over consecutive windows which are then turned into symbolic sequences. The combination of a Naïve Bayes GM with a first order Markov chain GM, outperforms conventional discriminating approaches using SVMs and crisp rules when a threshold of normality is set to pH equal to 7.15, achieving a sensitivity of 60.9% and a specificity of 81.7%. The use of symbolic representation compensates for the high computational cost related to GMs. In [19] a combination of a Genetic Algorithm (GA) for feature selection, with three different classifiers is used for the discrimination of normal and pathological cases. Cases with pH < 7.05 are considered pathological while cases with 7.27 < pH < 7.33 are considered normal. The GA with an SVM classifier with Radial Basis Function (RBF) kernels, regularized using the Bayesian information criterion (BIC), outperforms all other combinations achieving sensitivity equal to 83.02% and specificity equal to 66.03%. An ensemble/committee of ANNs is trained in [21] using normal and pathological cases that are defined slightly different between training and testing data sets (training: pathological pH < 7.1, normal 7.27 < pH < 7 .33 – testing: pathological pH < 7.1, normal 7.22 < pH < 7.27). Principal Component Analysis (PCA) is applied to reduce the dimension from 47 to just six and increase computational efficiency. A sensitivity of 60.3% and a specificity of 67.5% are reached.

It must be noted that that the aforementioned works use different databases, which in turn differ in many parameters: different size (40–500), different fraction of pathological cases, varying time until delivery, and use of different parts of the FHR signal for analysis (e.g. with or without the second stage of labor). More importantly these works use different pH thresholds while some of them use more than one threshold to define the classes’ boundaries. In the present study a single threshold is used. This threshold is set to 7.05, which is the setup used in the majority of technical papers on CTG classification [1, 27–30], because it provides sufficient compromise between the amount of pathological cases that are considered and the amount of complications related to the health status of the fetus [31–33].

More specifically, this study presents the results of FHR classification on the unique open access intrapartum CTU-UHB CTG database [33]. Both the involved set of features, which covers different domains, and the classification algorithm, which is based on a computational efficient variant of SVMs, could be considered to be among the current state of the art methods. A two stage feature selection procedure is used, which significantly reduces the number of involved features, further increasing the computational efficiency of the approach. To the best of our knowledge this is the most extensive testing performed on this data set and as such the achieved results can be used as a benchmark for the evaluation of other features and/or other classification algorithms, tested on this database. For comparison reasons and in order to check whether the use of the aforementioned advanced data processing methods offer an advantage over simpler approaches, the proposed method is also tested against a simpler classification scheme.

The rest of the paper is structured as follows: Section 2 describes briefly the methods employed in this work, from signal preprocessing and feature extraction to classification. Section 3 presents the results along with a discussion about the effect of the number of selected features and Section 4 concludes the paper with some directions for possible future research.

## Materials and methods

In this work the newly released CTU-UHB CTG database [33] is used. The proposed method classifies all the recordings using primarily the FHR signal and consists of the following steps: FHR preprocessing; Feature extraction; Feature ranking / selection and Classification. Matlab 2012b (The Mathworks, Inc.) is used to analyze the data. In the rest of this section all the involved steps are presented along with a description of the CTU-UHB CTG dataset.

### Data set

The open access CTU-UHB CTG database [33] consists of 552 records, which is a subset of 9164 intrapartum CTG recordings acquired between the years 2009 and 2012 at the obstetrics ward of the University Hospital in Brno, Czech Republic. All women signed informed consent and the study for the data collection was approved by the Institutional Review Board of University Hospital Brno. All CTG recordings and clinical data were anonymized. For this study the last 30 min of the 1st stage of labor are selected.

Clinical parameters were used to achieve as consistent a database as possible: Only fetuses with more than 37 completed weeks of gestation and singleton pregnancies were included. All fetuses with known intrauterine growth restriction, fetal infection and fetuses with congenital malformations were excluded. Only recordings that ended less than 20 min (median 5 min) before delivery were selected for the database. The gap between the time of end of the actual CTG signal and the time of birth in the form of mean (min, max) was 2.70 min (0, 29); the length of the first stage of labor was 225 min (45, 648); and the length of the second stage of labor was 11.87 min (0, 30).

**Fig. 1:**
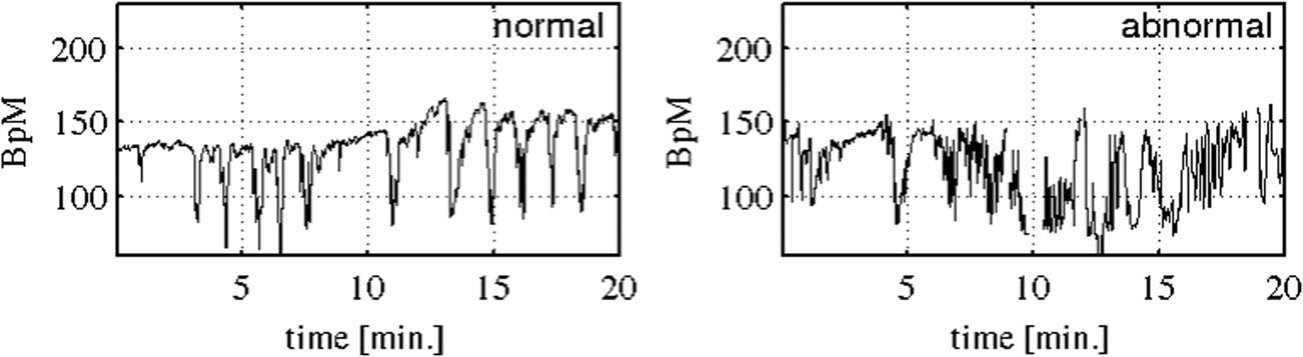
Typical FHR records for normal and abnormal cases. As it can be seen the FHR is a very irregular signal, which requires high degree of expertise to be correctly interpreted

From the 552 recordings, 44 of them have a pH value lower or equal to 7.05, which is the border line selected for defining the two classes in this study. Therefore, these 44 cases constitute the abnormal class while the rest 508 constitute the normal class, cf. Figure 1 for sample records. More details about the database and its construction can be found in [33].

### FHR preprocessing

The FHR signals were obtained either directly using a Doppler ultrasound (US) probe placed on mother’s abdomen, or from direct electrocardiogram (DECG) measured internally by a scalp electrode attached to the fetal scalp. Due to the acquisition method, FHR can be “contaminated” by spiky artifacts (impulsive noise) or contain periods where the FHR is zeroed. Spiky artifacts in the FHR as well as missing values can reflect on the values of the extracted features. Therefore a simple artifact rejection scheme is employed: first, extreme (not physiological) values (> 200 bpm and <50 bpm) are removed as in [9]; second, Hermite spline interpolation is applied to fill the gap of missing values [34]. We must note that long gaps (> 15 s) are not included in the subsequent feature extraction process. Despite its simplicity, this kind of artifact removal scheme is an established preprocessing step before further analysis can take place [35], even though more elaborate techniques have been proposed over the past years [36, 37].

On average 13.85% of the total duration of the 30 min segment consists of noisy data (artifacts including extreme values and missing data in less than 15 s gaps) with a minimum value of 0% and a maximum of 49%. Therefore, on average, the data set could be considered relatively stable, since in general noisy/missing data can amount to about 20%–40% of the total data length.

### Feature extraction

Feature extraction is probably the most important step in any classification problem, since an informative set of features makes the subsequent classification stage a much easier process. In this work a mixture of features is utilized. In total 54 features are used – coming from 21 basic features by varying some internal parameters, which are presented in summary in Table 1.

**Table 1:** Features used in presented work

Feature set	Features	Parameters
FIGO-based	Baseline, number of accelerations number of decelerations Δ_*total*_	Mean, standard deviation
Time domain	STV, STV-HAAN [38], STV-YEH [39], Sonicaid [40], SDNN, Δ [10], LTI-HAA [38]	
Frequency domain	energy03 [10] energy04 [41]	LF, MF, HF, LF/HF, VLF, LF, MF, HF, LF/(MF + HF)
Non-linear domain	FD_Variance [42], FD_BoxCount, FD_Sevcik [43] FD_Higuchi [44], DFA [45],	
	ApEn, [46] SampEn [47]	*r* = {0.15, 0.2}, *m* = {2,3}
	LZC [48]	
	Poincare	SD_1_, SD_2_

Abbreviations as follows: STV Short Time Variability, LTI Long Term Irregularity, Δ delta value, Δ_*total*_ the total value of Delta (long term variability defined in the FIGO guidelines), SDNN standard deviation of the NN interval, LF Low Frequency, MF Movement Frequency, HF High Frequency, VLF Very Low Frequency, ApEn Approximate Entropy, SampEn Sample Entropy, LZC Lempel - Ziv Complexity, FD Fractal Dimension, DFA Detrend Fluctuations Analysis, SD_1_ and SD_2_ Standard Deviation from Poincaré plot

Different feature domains represent different points of view of the CTG, ranging from FIGO-based features that try to emulate the information extractable by eye, to time domain features that are very understandable to clinicians yet impossible to see by naked eye, to more complex feature domains, which quantify the signal using frequency and nonlinear analysis tools. These latter approaches are well established for the analysis of adult’s HRV and are expected to perform well also in the case of FHR. All features are quite common in FHR analysis studies and have been already described in other publications [13, 18, 49] and cover the following areas:

- FIGO-based features: baseline, number of acceleration/deceleration, and long term variability. In this work for the extraction of the FIGO-based features, which describe the macroscopic properties of the FHR, the algorithms proposed in [50] are used.
- Time domain features: quantifying Short Term Variability (STV) and Long Term Irregularity (LTI).
- Frequency domain: energy in different frequency bands. These features are believed to capture the balance of behaviour of the two autonomic nervous system branches. A non-parametric Welch periodogram was used for the power spectral density (PSD) estimation (parameters: Gaussian-like window of size 1024 samples and 80% overlap). The energy in frequency bands was computed using [10, 41].
- Nonlinear domain: Fractal dimensions, Detrend Fluctuations Analysis (DFA), Entropy measures, Lempel-Ziv complexity, and Poincaré plot (embedding of RR_*n*_ intervals vs. RR_*n+1*_ (dimension *m* = 2, time delay *τ* = 1). All these features try to quantify the complexity of the signal under investigation.

### Feature selection

Usually, the step of feature extraction creates a large number of features. However as in almost all classification problems, not all of the extracted features are necessary for the classification task at hand. This happens because some of the features may convey overlapping information or even not be as informative as expected. Therefore a feature selection stage, or a dimensionality reduction stage, is usually involved before the application of the final classification algorithm [51]. This stage significantly decreases the time required for building/training a classifier, increasing therefore computational efficiency, while at the same it might improve the generalization capability of the classifier [51].

To put it more formally, the task of feature selection, for classification, can be described as follows: given an initial set of *N*_*F*_ features, select a subset of *j* features, where *j* <  < *N*_*F*_, retaining as much as possible of their class discriminatory information. The choice of a suitable subset of the features can allow the classifier to reach a near-optimal performance, which is a key step for any machine learning algorithm.

Feature selection algorithms can be divided into three general categories [52, 53]: filters, which do not require a learning algorithm, wrappers, which use a classification algorithm as part of the selection procedure and embedded methods, where the feature selection takes place at the same time of the classifier building, with each one of these methods having its pros and cons.

In this work, a hybrid approach is used, consisting of a filtering stage followed by a stage where eye-inspection (based on “experts” feedback) is used for the selection of the number of features that is to be included in the subset. Both steps of the feature selection process are described in the rest of this section.

The first step involves a “filtering” process during which, a measure is used to evaluate the effectiveness of each individual feature in predicting the class of each sample/example. The features are then ranked based on that measure: from the most helpful to the least helpful one. This method is very computational efficient and is preferred in conjunction with an advanced classifier or with subsequent fine tuning selection using e.g. a wrapper to further reduce the final set.

The feature selection process becomes more challenging for problems with class imbalance. One of the best ways to tackle the feature selection under class imbalance is to use the Receiver Operating Characteristic (ROC) curve and the corresponding value of the Area Under the ROC Curve (*AUC*) to rank the features, which is a measure that is immune to class imbalance [54]. In [54] the *AUC* was approximated using a small number of trapezoids leading to a very fast implementation. In this work, since the number of cases is relatively small, a more precise estimation is used relying on the Mann-Whitney-Wilcoxon two-sample statistic [55, 56]:

1 $$ {U}_1={n}_1{n}_2+\frac{n_1\left({n}_1+1\right)}{2}-{R}_1, $$

2 $$ AUC=\frac{U_1}{n_1{n}_2}, $$

where *n*_1_ is the sample size of the examples belonging to class 1, *n*_2_ is the sample size of the examples belonging to class 2 and *R*_1_ is the sum of the ranks from the samples of class 1.

**Fig. 2:**
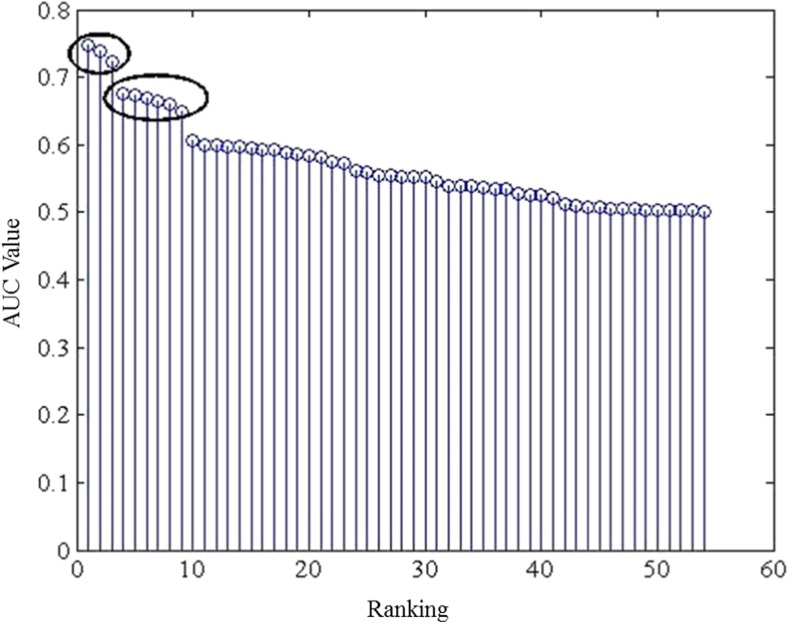
The *AUC* values of all 54 features for a random training sample. Each one of the two “clusters” of features with higher *AUC* values are marked with an ellipse. The first cluster contains, ranking from most important to the least important: energy at the VLF, energy at the LF [11], and SD_2_ of Poincaré plot. The second cluster contains, ranking from most important to the least important: ApEn *r* = 0.2 , *m* = 2, ApEn *r* = 0.15 , *m* = 2, LF/HF, SampEn, STV-HAAN, energy at the LF [41]

After the ranking stage, a visual inspection of the features’ ranking is performed. Features are plotted in descending order based on the *AUC* value that is estimated by leaving out each time a randomly selected positive example (a pathological case with *pH* ≤ 7.05) and a dozen of randomly selected negative examples (normal cases with pH > 7.05). The resulting pattern is consistent among the different trials having two distinct “clusters” of features consisting of three and six features. These clusters have much higher (individual) predictive value compared to other features, cf. Figure 2. Based on these observations, three different input feature sets are tested: a) the three individually “best” features (energy at the VLF [41], energy at the LF [10], SD_2_ (Standard Deviation of points along the line y = x of a Poincaré plot), b) the nine highest ranked features (the first three plus next six (ApEn *r* = 0.2 , *m* = 2, ApEn *r* = 0.15 , *m* = 2, ratio of energies in LF and High Frequency (HF) bands (LF/HF) [10], SampEn, STV [38], energy at the LF band [41]) and c) all 54 features.

### Classification using least squares support vector machines (LS-SVMs)

As it was described in the previous section, an *AUC* based filter selection scheme is applied to reduce the number of features and select those having a noticeable impact from the rest. However, by this setting the correlation between features is not considered. Therefore SVMs, a classification paradigm that is not affected so much from the presence of correlated inputs, is selected [57] to perform the categorization/decision task. More specifically the Least Squares version of the SVMs (LS-SVMs) [58] is chosen due to the much faster training time required when moderate size problems are tackled (the computational complexity for a naïve implementation is *O*(*N* ^3^) where *N* is the size of the training set, but other faster approaches exist [59]).

SVM classifiers map the data into a higher dimensional space and then an optimal separating hyper-plane is constructed. Given a set of *N* training samples {(*x*_*i*_, *y*_*i*_), *i* = 1,  … , *N*}, where ${x}_i\in {\mathbb{R}}^{N_f}$ (*N*_*f*_ being the dimension of the input space) and the corresponding labels *y*_*i*_ = {+1, −1}, the support vector method aims to construct a classifier of the form:

3 $$ {w}^T\varphi \left({x}_i\right)+ b\ge +1,\mathrm{if}\;{y}_i=+1 $$

4 $$ {w}^T\varphi \left({x}_i\right)+ b\le -1,\mathrm{if}\;{y}_i=-1 $$

or equivalently

5 $$ {y}_i\left[{w}^T\varphi \left({x}_i\right)+ b\right]\ge 1, i=1,\dots, N, $$

where *φ*(⋅) is a nonlinear function which maps the input space into a higher dimensional space, with *b* a scalar and *w* an unknown vector with the same dimension as *φ*(⋅).

For the standard SVM algorithm the following optimization problem is formulated:

6 $$ \begin{array}{l}\kern2em {min}_{w,\mathrm{b},{\xi}_k}{F}_1\left( w,{\xi}_i\right)=\frac{1}{2}{w}^T w+ C\sum_{i=1}^N{\xi}_i\hfill \\ {}\mathrm{Subject}\ \mathrm{to}:{y}_i\left[{w}^T\varphi \left({x}_i\right)+ b\right]\ge 1-{\xi}_i, i=1,\dots, N\hfill \\ {}\kern6em {\xi}_i\ge 0, i=1,\dots, N\hfill \end{array} $$

where, *ξ*_*i*_ s are slack variables that allow misclassifications in the set of inequalities (e.g., due to overlapping distributions). The positive real constant *C* is considered as a tuning parameter in the algorithm. For the case of the LS-SVM classifier, instead of Eq. (6), the optimization problem is formulated as in [58]:

7 $$ \begin{array}{l}\kern3em { \min}_{w, b, e}{F}_2\left( w, e\right)=\frac{1}{2}{w}^T w+\frac{1}{2}\gamma \sum_{i=1}^N{e}_i^2\hfill \\ {}\mathrm{Subject}\ \mathrm{to}:{y}_i\left[{w}^T\varphi \left({x}_i\right)+ b\right]=1-{e}_i, i=1,\dots, N,\hfill \end{array} $$

where *e*_*i*_ is an error variable and *γ* is a regularization parameter.

The above formulation leads to the construction of a decision function of the form:

8 $$ y(x)=\mathit{\operatorname{sign}}\left(\sum_{i=1}^N{a}_i{y}_i K\left({x}_i, x\right)+ b\right), $$

which implies that every training data point is a support vector. *K*(⋅, ⋅) is a kernel function that implicitly performs the mapping form the input to the high dimensional feature space and *a*_*i*_ are the Lagrange multipliers.

In this work the RBF kernel is used:

9 $$ K\left({x}_i,{x}_j\right)= \exp \left(\frac{-{\left\Vert {x}_i-{x}_j\right\Vert}_2^2}{\sigma^2}\right), $$

where *σ* is the spread parameter of the RBF kernel.

The above formulation works fine in the case of well-balanced classes. However for cases with imbalanced distribution between the two classes, a mechanism for compensating for this is needed [60]. One of the simplest methods relies on subsampling the majority class. However this might discard some of the patterns that lie on the decision boundary. To avoid this problem, a second approach based on the calculation of unequal costs for the two classes is used [18, 60]. In the case of LS-SVMs, the formulation becomes.

10 $$ \begin{array}{l}\kern2em {min}_{w, b, e}{F}_2\left( w, e\right)=\frac{1}{2}{w}^T w+\frac{1}{2}\gamma \sum_{i=1}^N{v}_i{e}_i^2\hfill \\ {}\mathrm{Subject}\;\mathrm{to}\;{y}_i\left({w}^T\varphi \left({x}_i\right)+ b\right)=1-{e}_i, i=1,\dots, N,\hfill \end{array} $$

where *v*_*i*_ is given by [61]:

11 $$ {v}_i=\left\{\begin{array}{cc}\hfill \frac{N}{2{N}_p}\hfill & \hfill if\;{y}_{\iota}=+1\hfill \\ {}\hfill \frac{N}{2{N}_{\mathrm{N}}}\hfill & \hfill if\;{y}_{\iota}=-1\hfill \end{array}\right\}, $$

with *N*_*p*_ and *N*_*N*_ representing the number of “positive” and “negative” training samples respectively. In this work, a fine tuning of the ratio between the two penalty factors is sought during the parameter selection process.

For the LS-SVM implementation, the LS-SVMlab toolbox is used (http://www.esat.kuleuven.be/sista/lssvmlab/).

## Results

Due to the small number of abnormal cases (44 in total) a 44-fold stratified cross-validation is used for performance estimation. The employed cross validation consists of an outer and an inner loop. In the inner loop the LS-SVM parameters are tuned while in the outer loop the performance is estimated. The number of folds is set such that the best exploitation of the limited number of “abnormal” cases is achieved. More specifically for each fold, one case belonging to the abnormal set and 12 (or 11) cases belonging to the normal set are used for testing, leaving 43 abnormal and 496 (or 497) normal cases reserved for training. The training set is normalized so that each feature has mean value equal to zero and standard deviation equal to one. The learned transform is then applied to the testing data.

**Fig. 3:**
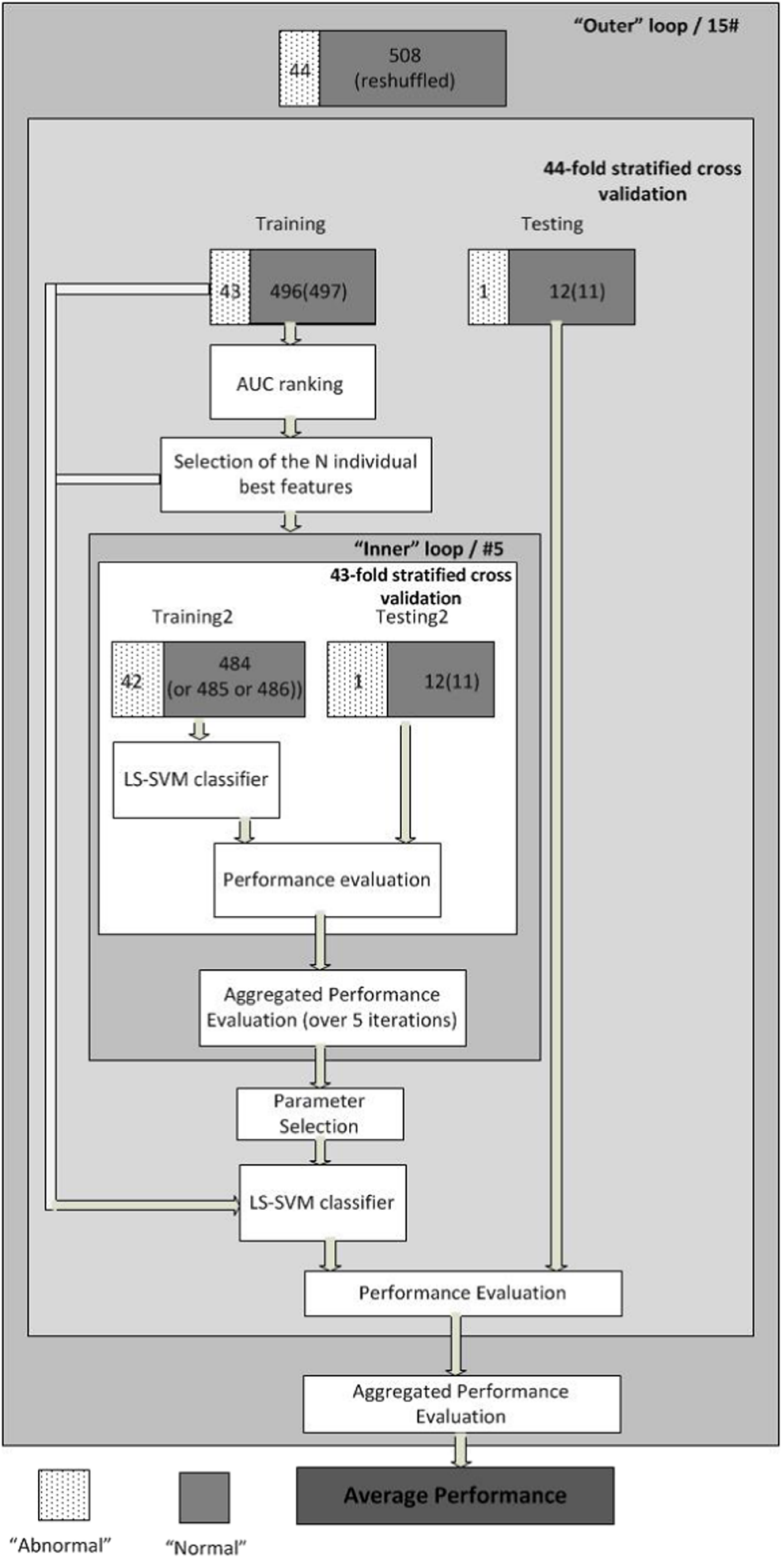
The overall procedure

Before testing the LS-SVM the involved parameters are tuned (i.e. *σ*, *C*, and the imbalance factor) using the training data and a 43-fold stratified cross validation procedure. This inner-loop procedure is repeated five times and each time a reshuffling of the normal cases takes place ensuring that for each one of the five repetitions each abnormal case is never matched with the same 12 (or 11) normal cases. The whole evaluation procedure is repeated 15 times, each time reshuffling the samples corresponding to the normal cases. Figure 3 depicts the whole process.

The applied tuning process is used in order to select good-near-optimal parameter values subject to a specific criterion / performance measure. In general, all classification measures can be estimated using the elements of the confusion matrix (Table 2), with sensitivity and specificity being among the most commonly reported values for medical settings.

**Table 2:** A general confusion matrix for a binary problem

	Predicted as positive	Predicted as negative
Actual positive	True positives (*TP*)	False negatives (*FN*)
Actual negative	False positive (*FP*)	True negatives (*TN*)

Some of the most common performance measures are:

- the overall accuracy:

12 $$ Accuracy=\frac{TP+ TN}{TP+ TN+ FP+ FN}, $$

the True Positive Rate (*TP*_*rate*_) also known as Sensitivity or Recall:

13 $$ {TP}_{rate}=\frac{TP}{TP+ FN}, $$

the True Negative Rate (*TN*_*rate*_) also known as Specificity:

14 $$ {TN}_{rate}=\frac{TN}{TN+ FP}, $$

and the Positive Predictive Value (*PPV*) also known as Precision:

15 $$ PPV=\frac{TP}{TP+ FP}. $$

Conventional overall accuracy, is not suitable for problems with high imbalance between the classes, because with imbalanced datasets it leads to the adoption of classifiers that may completely neglect the minority class [62] (in the current case a classifier that assigns everything to the negative class would have an accuracy value of 508/552 or 92.3% but would be practically useless). In order to avoid that, four alternative measures of classification performance are used, which manipulate differently the entries of the confusion matrix:

- Balanced Error Rate (*BER*):

16 $$ BER=\frac{1}{2}\left(\frac{FP}{FN+ TP}+\frac{FN}{FP+ TN}\right), $$

b) Geometric mean (*g-mean*):

17 $$ g- mean=\sqrt{TP_{rate}\cdot {TN}_{rate}}, $$

c) Harmonic mean (*F-measure*):

18 $$ F- measure=\frac{2}{\frac{1}{TP_{rate}}+\frac{1}{PPV}}, $$

d) Matthews Correlation Coefficient (*MCC*):

19 $$ MCC=\frac{TP\cdot TN- FP\cdot FN}{\sqrt{\left( TP+ FP\right)\left( TP+ FN\right)\left( TN+ FP\right)\left( TN+ FN\right)}}. $$

From the four measures, only the *BER* has an inverse relationship to performance (smaller values correspond to better performance). For all the others the higher the value the better the classifier is. *BER* is a common measure of performance in the case of imbalanced data sets [54]. *g-mean* has also been used in the context of FHR classification [18] and it is often employed in the case of imbalanced data sets. In the case of the *F-measure*, the simplest form is selected, which corresponds to estimating the harmonic mean of precision (*PPV*) and recall (*TP*_*rate*_) which usually leads to balanced values between precision and recall [63]. Finally, *MCC* is another measure not affected by the different size of the two classes [64].

**Fig. 4:**
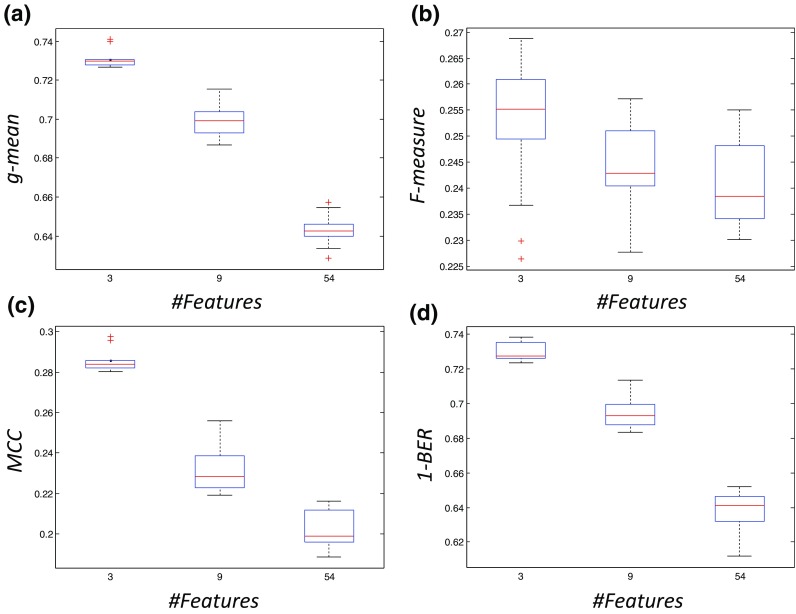
Performance measures: *g-mean*, *F-measure*, *MCC* and 1-*BER* (**a**), (**b**), (**c**) and (**d**) respectively for the different number of input feature sets

The performance for the different input feature sets and for the different measures are summarized in Fig. 4, where instead of the *BER*, 1- *BER* is included so that higher values correspond to better performance as in the other three measures. It should be noted that for each one of the reported measures, the same criterion is used during the tuning process. In other words the *g-mean* values shown in Fig. 4a correspond to an LS-SVM whose parameters were selected using *g-mean* as the tuning criterion etc.

**Fig. 5:**
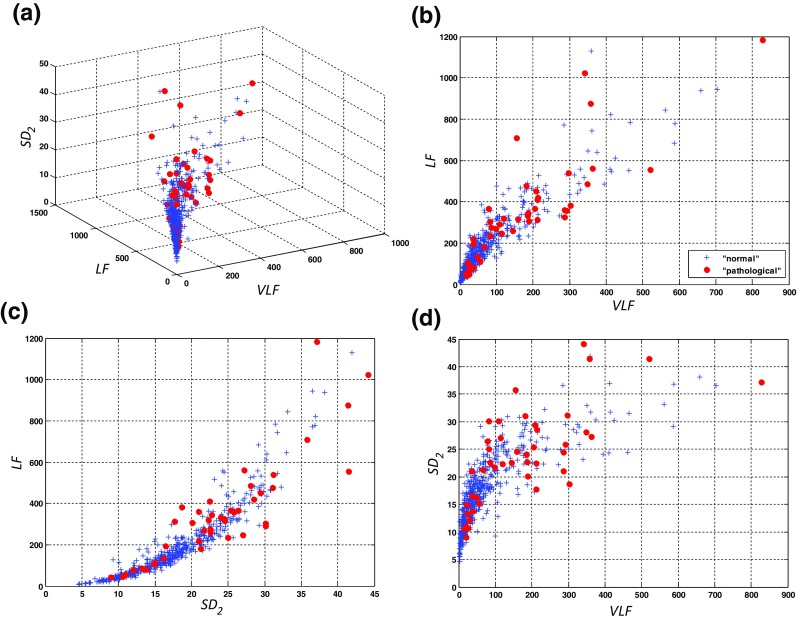
Visualization of the top three ranked features VLF – Energy at the VLF band [41], LF - Energy at the LF band [10], SD_2_. **a** 3D scatter using all three features, **b** – **d** features pair-wise 2D scatter plot

**Fig. 6:**
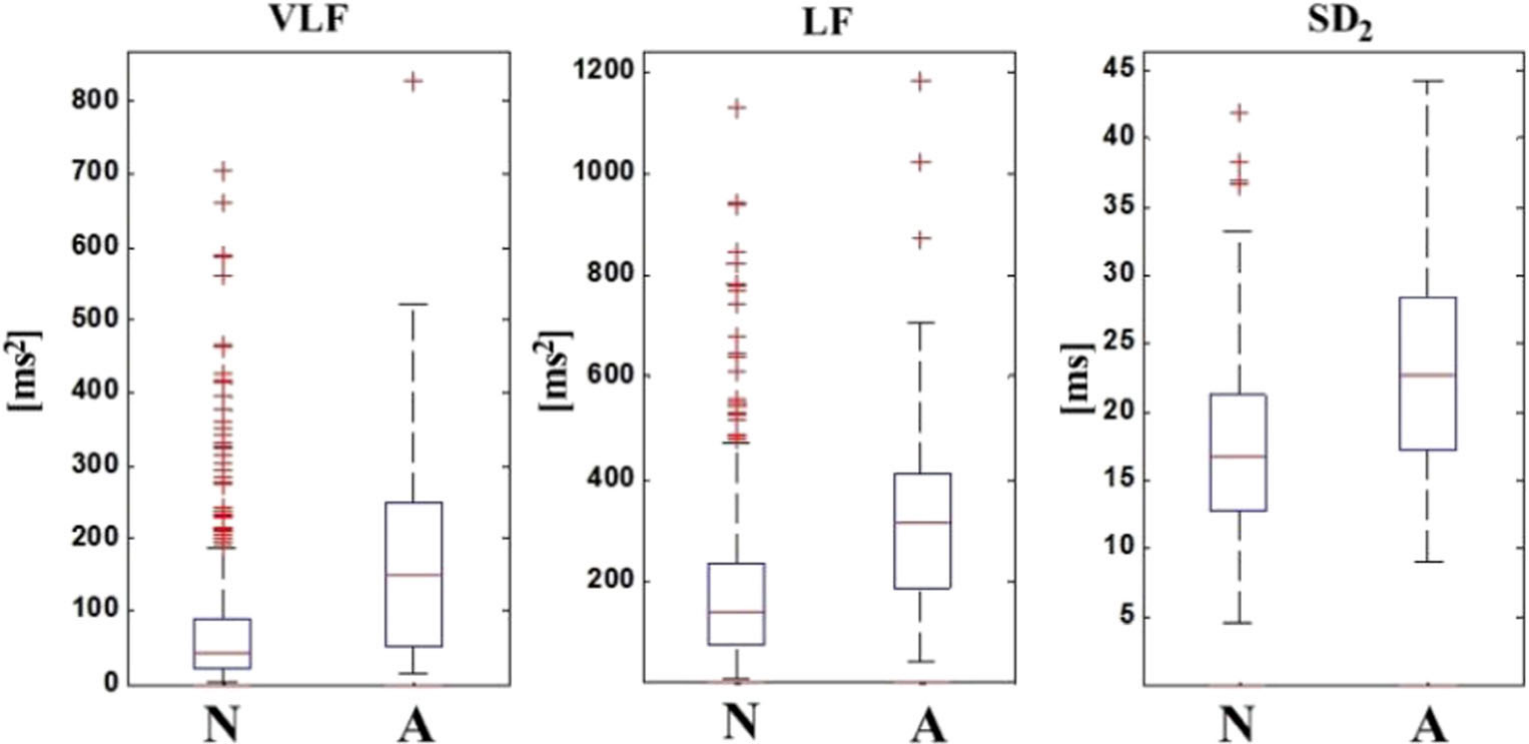
Visualization of the top three ranked features VLF – Energy at the VLF band [41], LF - Energy at the LF band [10], SD_2_. (*A*: Abnormal, *N*: Normal)

The average values are reported in Table 3. From Fig. 4 it is evident that the best performance for all four different measures is achieved using only the three individually best features (highlighted in bold) and the performance shows a decreasing trend with the addition of more features (Note: this statement holds only for this specific ranking of the features and the specific three feature levels (3–9-54)). Figure 5 shows the feature space for the three top ranked features, highlighting the non-separable nature of pH based classes. This is further supported by Fig. 6 showing the non-separable nature of normal and abnormal cases for the three top ranked features (VLF, LF, and SD_2_) (Note: The difference in median between normal and abnormal cases is statistically significant for all three features (*p* < 0.05, Wilcoxon rank sum test)). While it is difficult to tight these features to a precise underlying physiological mechanism, it is clear that VLF and SD_2_ correlates with frequency of FHR decelerations and LF is associated with neural sympathetic activity [10, 32, 41]. In terms of sensitivity and specificity, the results are summarized in Table 4, where each row corresponds to the results of the application of LS-SVMs having as inputs three features and tuned using the criterion listed in the first column. The aggregated confusion matrices for the case of the input set with only 3 features are presented in the appendix. From Table 4 it can be seen that *F-measure* seems to lead to a different configuration of the LS-SVM while the other three criteria lead to classifiers with similar performance*.*

**Table 3:** Average performance for the different input feature sets

#Features	1*-BER*	*g-mean*	*F-measure*	*MCC*
3	**0.7305**	**0.7294**	**0.2523**	**0.2850**
9	0.6997	0.6949	0.2442	0.2318
54	0.6431	0.6388	0.2406	0.2025

**Table 4:** Average sensitivity and specificity values, for the three best input features, under different tuning criteria

Tuning criterion	Sensitivity	Specificity
*BER*	0.6848	0.7768
*g-mean*	0.6879	0.7735
*F-measure*	0.7212	0.6530
*MCC*	0.6848	0.7768

As also explained in the next section, comparison of the variety of methods found in the literature is not straightforward. Therefore in order to further validate the utility of the proposed approach and more specifically the approach that utilizes only the top three features, a comparison with a more conventional classification scheme is performed. This scheme involves a dimensionality reduction stage, a means to compensate for the class imbalance, and a simple classifier. However, this time the dimensionality reduction stage is not performed through feature selection but via the use of PCA [51], the imbalance compensation is performed using the Synthetic Minority Oversampling TEchnique (SMOTE) [65] and the classifier is a linear one, the Minimum Mahalanobis Distance Classifier (MMDC) [51].

PCA has been used regularly in classification approaches involving the FHR [18, 21] and even though it is a linear unsupervised technique, it can be very competitive to more advanced schemes when it comes to applications of real life data [66]. SMOTE is also a method that has been widely used in FHR analysis, since in this field normal cases dominate existing datasets. The MMDC is a linear method which despite its simplicity can perform “embarrassingly” (for more advanced schemes) well when applied to real life data [67]. Another appealing property of the MMDC is that it is a parameter free method. However even for this simple classification scheme and despite the fact the MMDC does not have any parameters to be tuned, the other two stages need tuning: selection of the number of retained PCs and the amount of oversampling of the abnormal class for SMOTE.

**Fig. 7:**
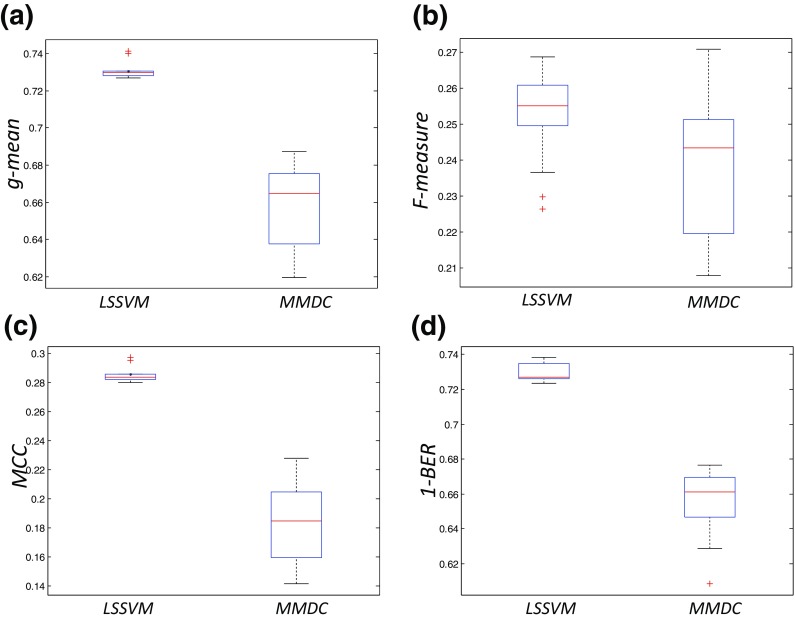
Performance measures: *g-mean*, *F-measure*, *MCC* and 1-*BER* (**a**), (**b**), (**c**) and (**d**) respectively for the case of the LSSVM classifier having as input the aforementioned three features, against the case of the MMDC based approach

As in the case of the LS-SVM scheme, a grid search is performed following the same procedure as described above. The same four performance measures are involved and the results are depicted in Fig. 7, against the results achieved using the more elaborate classification scheme using only thee input features. From Fig. 7, it is evident that the extra effort for developing the proposed classification scheme is indeed beneficial.

## Discussion

In this work, a large set of available (and commonly used) features that are extracted from open access CTU-UHB CTG database is examined for classification. These features originated from different domains in order to cover as much as possible of the information contained in FHR that could be associated with delivery outcomes. The delivery outcome is quantified using umbilical artery pH. The analysis shows that the use of only three features combined with a powerful, yet computational efficient classifier, can achieve sensitivity and specificity values that are close or above 70%. The balanced nature of the results is reached by taking into account the imbalanced nature of the problem during the training phase of the LS-SVM (using unequal costs for the two classes, Eq. (10)).

Even though comparison to other published works is almost impossible the proposed approach is comparable or even outperforms the published literature. Compared to [34], where a similar approach was pursued with a filter selection (RELIEF algorithm) and a boosted mean prototype classifier, the results are better (sensitivity equal to 64.1% and specificity equal to 65.2%). Moreover the current approach reaches higher performance values using a smaller number of features, thus improving computational efficiency.

Compared to the results presented in [29], where a sensitivity equal to 57% and a specificity of 97% is reported, the current work achieves better sensitivity but worse specificity. However, these findings should be viewed with caution, since in [29] a different data set is involved. Regarding the computational complexity the system employed in [29] has a very fast inference mechanism based on well-established medical criteria [68]. Our method on the other hand, requires training, but once trained the response of the model is very fast, especially since it requires the extraction of only three features.

Regarding other works that use the CTU-UHB CTG database for pH based classification, as in [69, 70], direct comparison is again not possible since higher a threshold is used (pH threshold equal to 7.2) in combination with other criteria (Note: pH is a logarithmic measure and different thresholds can lead to dramatically different stratification of the data leading to completely different classification performance), while in [69] the approach is more of an exploratory nature. Finally, compared to the simpler scheme using linear methods and SMOTE for compensating the data imbalance, the proposed approach is much more effective. Table 5 summarizes the performance of the aforementioned works along with the involved feature space, which however should be treated with caution since different criteria and different datasets are involved.

**Table 5:** Summary of recent approaches using pH as a means to class formation

Reference	Feature space	Sensitivity	Specificity	Criterion
Xu et al. 2014 [19]	Baseline, STV, LTV, Acceleration duration, Auto-mutual information, Approximate entropy, Sample entropy, (STD/mean)^2^, Phase rectified signal averaging	83.02%	66.03%.	pH < 7.05 and 7.27 < pH < 7.33
Georgieva et al. 2013 [21]	Signal quality, Baseline, Signal stability index, Minimal expected FHR value, #decelerations, Onset slope of the decelerations, Gestation (weeks), Maternal temperature, Parity, Meconium staining, Epidural/Spinal analgesia, Sex	60.3%	67.5%	pH < 7.10 and 7.27 < pH < 7.33
Dash et al. 2014 [23]	A single discrete valued feature that combines variability, accelerations and decelerations	60.9%	81.7%.	pH ≤ 7.15
Costa et al. 2009 [29]	Reduced long-term variability, repetitive decelerations, tachycardia, decelerations, reduced STV, reduced variability, ST event	57%	97%	pH ≤ 7.05
Spilka et al. 2013 [34]	Baseline, STV, LTV, Accelerations, Decelerations, Energy in frequency bands, Approximate and sample entropy, fractal dimension, SD_1,_ SD_2_	64.09%	65.2%	pH ≤ 7.05
Rotariu et al. 2014a [69]	MF/(LF + MF + HF), HF/(LF + MF + HF), MF⁄HF^a^	96%	87.6%	pH < 7.2 and BDecf >8 mmol/L
Rotariu et al. 2014b [70]	Accelerations, Decelerations, Prolonged decelerations	73.2%	88.2%	pH < 7.2 and Apgar <6
Current work (MMC)	VLF, LF, SD_2_	68.48%	77.68%	pH ≤ 7.05
Current work (F-measure)	VLF, LF, SD_2_	72.12%	65.30%	pH ≤ 7.05

^a^Low frequency LF (0.03–0.07 Hz), mid-frequency MF (0.07–0.13 Hz), and high frequency HF (0.13-1 Hz)

The performance of the proposed method seems to be in agreement with the findings of [32], where a *g-mean* equal to 0.7 is anticipated for large datasets. However more research is needed before a conclusion can be reached on the limitations of an approach based solely on FHR processing and pH based class formation. Moreover, Fig. 5 clearly shows quite an overlap between the two classes indicating that a perfect classification, in the current setting, may be impossible. Therefore new features, including also information coming from the UC signal [71], and/or better algorithms for classification are needed. This work can therefore act as a benchmark for the evaluation of new features and algorithms, since it only requires the extraction of three features and the use of a computational method, the training of which can be done really fast.

## Conclusion

In this work a method for the evaluation of FHR is proposed and tested using the open access CTU-UHB CTG database with promising results. For the specific setting, a minimal set of three input features seems to produce the best results in terms of performance measure developed for imbalanced data sets. These results are comparable to those achieved by other methods presented in the literature, and outperform a simpler classification scheme, which is used as a “base” measure to validate the use of advanced data processing techniques. However the lack of standardization makes it impossible to have a more formal comparison. The proposed approach, being the most complete experimental study so far, could be used as a benchmark for future studies involving the CTU-UHB CTG open access database.

The results also seem to confirm the findings of [32] that reported difficulties in obtaining high classification performance using FHR recordings and pH based classes on large datasets. Therefore, either other source of information should be seek, such as the inclusion of the Maternal Heart Rate (MHR) [72], ST analysis [29], or other clinical information as part of the feature set, and/or alternative labeling process should be considered, keeping also in mind that it is not natural to have a simple separating line (pH based) between the normal and abnormal (pathological) FHR groups. Toward the latter, a model for aggregating experts’ opinion has been recently proposed based on the Latent Class Analysis (LCA) [73–75]. In future work we plan to investigate a hybridization of both approaches in hope of developing more reliable decision support tools for the interpretation of CTG recordings.

## Appendix

**Table 6:** Aggregated confusion matrix for the case of three features and 1-*BER* criterion

	Predicted as abnormal	Predicted as normal
Actual abnormal	452	208
Actual normal	1706	5914

**Table 7:** Aggregated confusion matrix for the case of three features and *g-mean* criterion

	Predicted as abnormal	Predicted as normal
Actual abnormal	454	206
Actual normal	1726	5894

**Table 8:** Aggregated confusion matrix for the case of three features and *F-measure* criterion

	Predicted as abnormal	Predicted as normal
Actual abnormal	476	184
Actual normal	2641	4979

**Table 9:** Aggregated confusion matrix for the case of three features and *MCC* criterion

	Predicted as abnormal	Predicted as normal
Actual abnormal	452	208
Actual normal	1701	5919
